# Gait Analysis for Early Detection of Motor Symptoms in the 6-OHDA Rat Model of Parkinson's Disease

**DOI:** 10.3389/fnbeh.2018.00039

**Published:** 2018-03-06

**Authors:** Jordi Boix, Daniela von Hieber, Bronwen Connor

**Affiliations:** Department of Pharmacology and Clinical Pharmacology, Centre for Brain Research, School of Medical Science, Faculty of Medical and Health Sciences, University of Auckland, Auckland, New Zealand

**Keywords:** gait analysis, Parkinson's disease, 6-hydroxydopamine lesion, substantia nigra pars compacta, midbrain dopamine neurons, motor deficiencies

## Abstract

Computer-supported gait analysis has proven to be effective for the comprehensive assessment of gait changes in rodent models of neurodegenerative and neurological disorders. However, full characterization of individual gait parameters is required for specific neurological or neurodegenerative disorders such as Parkinson's disease (PD). Gait disturbances in particular present as the most constraining set of symptoms in PD, finally depriving patients from most activities of normal daily living. In this study, we have characterized the gait pattern abnormalities observed in two rat models of PD: the medial forebrain bundle (MFB) 6-OHDA lesion model and the striatal 6-OHDA lesion model. Our data indicates significant changes in 21 different gait parameters in the MFB lesion cohort. We observed a steady decline in the overall walking speed and cadence, as well as significant alterations in the gait parameters stride length, initial dual stance, paw print position, step cycle, swing phase of the step cycle, stand index, phase dispersion, print length, and print area in at least one of the paws. These alterations correlated with the extent of tyrosine hydroxylase (TH) neuronal loss observed in this group. These alterations were detected as early as 1 week post lesion. In contrast, limited gait dysfunction was detected in the striatal lesion cohort related to the low level of TH neuronal loss detected in this group. In this study we have demonstrated that gait analysis is a reliable method for the detection of motor deficiencies in a MFB 6-OHDA lesion model of PD and may prove a clinically relevant, low impact method of testing functional impairment as early as 1 week post lesion.

## Introduction

In recent years, computer-supported gait analysis has proven to be effective for the comprehensive assessment of gait changes in rodent models for a range of neurodegenerative and neurological disorders (Vlamings et al., 2007; Chuang et al., 2010; Vandeputte et al., 2010; Hsieh et al., 2011; Wang et al., 2012; Westin et al., 2012; Parkkinen et al., 2013; Zhou et al., 2015; Baldwin et al., 2017; Kwon et al., 2017). However, proper validation and characterization of individual gait parameters is required for each specific neurological or neurodegenerative disorder. Parkinson's disease (PD) is a neurodegenerative disorder with progressive loss of dopaminergic (DA) neurons in the substantia nigra pars compacts (SNc) (Damier et al., 1999; Dauer and Przedborski, 2003). The degeneration of nigrostriatal innervation of the striatum and the subsequent reduced levels of DA in this area are responsible for the manifestation of the characteristic motor symptoms of PD (Ehringer and Hornykiewicz, 1960; Chuang et al., 2010). Severe motor deficiencies develop when a minimum of 30% loss of DA has been reached, represented by tremor at rest, bradykinesia, rigidity, loss of postural reflexes, flexed posture, and the freezing gait phenomenon (Braak et al., 2003; Fahn, 2003). These intractable symptoms, commonly summarized as Parkinsonism, dominate the clinical picture of PD and eventually lead to immobility, postural instabilities and an increased risk of injuries (Fahn, 2003; Frenklach et al., 2009; Kerr et al., 2010; Glajch et al., 2012).

A point often overlooked is that many PD patients present with an asymmetrical onset of symptoms where only one body-side is affected (Kempster et al., 1989; Plotnik et al., 2005; Djaldetti et al., 2006). It is important for the evaluation of any new treatment approach to use pre-clinical models that resemble this aspect. The 6-hydroxydopamine (6-OHDA) PD model, is produced by a unilateral intracerebral injection of the neurotoxin 6-OHDA and therefore dopaminergic cell death occurs in the midbrain on one side of the brain only and asymmetric motor symptoms can be elicited (Ungerstedt, 1968; Ungerstedt and Arbuthnott, 1970; Simola et al., 2007). The severity of symptoms in the 6-OHDA model depends on the site of injection and the dose of 6-OHDA used. In the “full lesion” model or medial forebrain bundle (MFB) model, injections of the toxin into the MFB cause an acute and near complete loss of DA neurons in the SNc with significant loss of striatal DA content (Faull and Laverty, 1969; Jeon et al., 1995). However, if the toxin is injected into the striatum, it reaches the SNc via retrograde transport along dopaminergic fibers and progressive degeneration of dopaminergic neurons can be observed with an overall milder pathology, known as the “partial lesion” or “striatal lesion” model (Sauer and Oertel, 1994; Przedbroski et al., 1995). In both models, the full extent of DA loss can take 3–4 weeks to complete (Faull and Laverty, 1969; Sarre et al., 2004). Lesions of the midbrain and their development over time can be assessed through investigation of changes in motor function. For this purpose, a wide range of behavioral tests have been developed, including the spontaneous exploratory forelimb use (cylinder test), the corridor test, the stepping test and drug-induced rotational analysis (Grealish et al., 2010; Bové and Perier, 2012; Boix et al., 2015). However, with these established methods it is often necessary to wait until the full extent of DA loss has occurred to observe a functional impairment. It is therefore advantageous to be able to identify reliable signs of functional impairment as early after lesioning as possible to allow for the assessment of potential therapeutic agents. Furthermore, despite their usefulness in the evaluation of DA depletion, none of the commonly used behavioral tests actually assesses symptoms that resemble those experienced by PD patients. Notably, gait disturbances have been reported to be the most common motor problems in PD patients and present themselves as the most constraining set of symptoms experienced by patients, finally depriving patients from most activities of normal daily living (Chuang et al., 2010). The aim of the present study therefore is to describe a proper methodological system to clearly identify which of the numerous gait analysis parameters are applicable to the characterization of gait disturbances in early stages of the 6-OHDA lesion rat models of PD.

## Materials and methods

### Subjects

Forty-seven adult male Wistar rats (starting weight 250–300 g, 8–11 weeks old; University of Auckland Vernon Jansen Animal Resources Unit, Auckland, New Zealand) were housed in a temperature and humidity controlled environment and kept on a 12/12 h reversed light/dark cycle with access to food and water *ad libitum*. Training and testing was performed during the dark phase of the cycle. All the experimental procedures were in compliance with the NZ Animal Welfare Act (1999) and in accordance with the University of Auckland animal ethics approval. Ethical number R001513 and SOP 001344/6.

### Surgical procedure

Animals were randomly allocated to one of four study groups either receiving a 6-OHDA injection into the striatum (Striatal cohort; *n* = 17), a 6-OHDA injection into the MFB (MFB cohort; *n* = 15) or a sham treatment (Sham striatum cohort; *n* = 6 and Sham MFB cohort; *n* = 9). Additional to the standard chow, animals received 5 grains of Cocopops® (Kellogg's) into their home cage daily for the first 2 weeks of the study to familiarize them with the taste.

Surgical level anesthesia was achieved using isoflurane (induction 5% isoflurane and 2.0 L/min O_2_; maintenance 2.5% isoflurane and 0.8 L/min O_2_). Anesthetized rats were placed in a stereotactic frame (Kopf, Tujunga, USA) before the rodents' heads were shaved and a burr hole was drilled. Rats allocated to the MFB lesion cohort received either an injection of 16 μg 6-OHDA (Sigma-Aldrich) dissolved in 3.2 μL of 0.2 mg/ml ascorbic acid in 0.9% sterile saline or vehicle injection (Sham) into the MFB of the right hemisphere, at the coordinates: 4.4 mm A/P, 1.1 mm M/L, and 8.0 mm D/V to Bregma (Paxinos and Watson, 1997). Rats allocated to the Striatal lesion cohort received the same concentration of 6-OHDA into the right striatum at the coordinates 0.1 mm A/P, 3.2 M/L, 5.0 mm D/V to Bregma (Paxinos and Watson, 1997). Sham operated animals received a 3.2 μL of 0.2 mg/ml ascorbic acid in 0.9% sterile saline. All injections were administered at the rate of 1 μL/min and the cannula was left in place for an additional minute to prevent backflow.

### Behavioral testing

Behavioral testing was performed in the order of lowest to highest impact on animals and with no more than two different tests per day.

**Figure 1:**
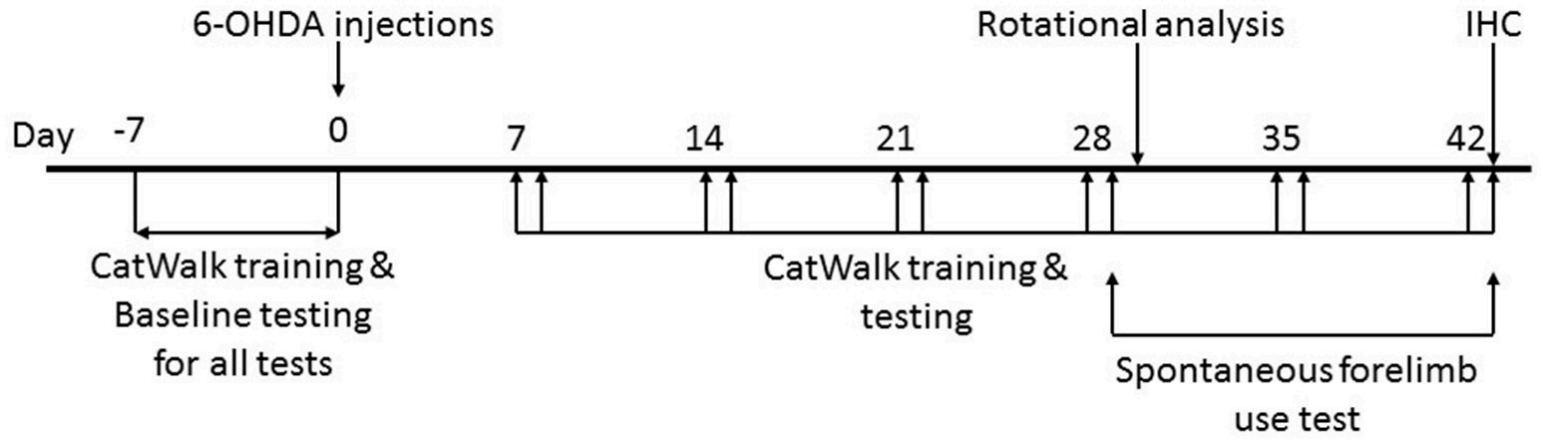
Timeline of the study. Animals were handled daily for 1 week prior to surgical intervention to make them amenable to human interaction. CatWalk™ XT training and baseline testing for CatWalk™ XT gait analysis, rotational analysis, and the spontaneous forelimb use test were conducted in the week preceding the 6-OHDA lesions. Behavioral testing was resumed on day 7 after the surgical intervention with training on the CatWalk™ XT on days 7, 14, 21, 28, 35, and 42 followed by acquisition of gait parameters with the CatWalk™ XT software on days 8, 15, 22, 29, 36 and 43. The spontaneous forelimb use test was conducted on day 29 and 43 after the CatWalk™ XT gait analysis. Rotational analysis was performed on day 30. All animals were sacrificed on day 43 after behavioral testing.

All tests were performed in red light conditions (LED 20 W) in sound attenuated, temperature and humidity controlled rooms (Figure 1).

#### CatWalk™ XT gait analysis

The gait of unforced moving rats was analyzed with the CatWalk™ XT system (Noldus Information Technology, Wageningen, The Netherlands) as described previously (Hamers et al., 2001; Vlamings et al., 2007). In an initial training period prior to 6-OHDA lesioning, rats were allowed to walk across the 70 cm long glass walkway for as many times as needed, but for a minimum of nine runs, in order to learn to walk in a straight line to the goal box positioned at the end of the walkway. A quantity of five grains of Cocopops® was placed at the entry to the goal box to increase motivation of animals to cross the walkway. Once trained, baseline testing followed prior to 6-OHDA lesioning and subsequent, weekly testing started 8 days after 6-OHDA lesioning and consisted of a minimum of three uninterrupted crossings (three runs). Each testing day was preceded by a training day on which animals were allowed to cross the walkway nine times, split up in three trials each with three runs and each trial separated by a 10 min long interval. This training was intended to re-familiarize the animals with the task and resulted in crossing the walkway without hesitation at a constant speed. Runs with any climbing, turning or any other interruptions were removed from the analysis. The CatWalk™ XT software was used to analyze runs that had at least three complete step cycles. Partial prints at the beginning or the end of the runs were removed from the analysis.

##### Gait parameters

All gait parameters examined in this study with the CatWalk™ XT system and showing significant differences are described below. Step cycle, swing phase, stand phase, stride length, print position, and cadence have all been reported previously to correlate with speed and have thus been normalized before being statistically analyzed (Batka et al., 2014). These parameters were normalized against the average speed of all animals by multiplication of each individual value with a normalization factor. The normalization factor F was calculated as follows:

$$\begin{array}{l} {\text{F}}_{{\text{n}}_{1}},{\text{n}}_{2},\ldots m_{1}\ldots =\frac{{\text{V}}_{{\text{n}}_{1},{\text{n}}_{2}},\ldots m_{1\ldots }}{average\;{\text{V}}_{m,n,\ldots }} \end{array}$$

*F*_*n*_1_,*n*_2_, …*m*_1,…__ representing the normalization factor for each test animal at a certain time point n, m,_…_ etc.

*V*_*n*_1_,*n*_2_,…*m*_1,…__ representing the individual speed at which each animal crossed the walkway at a certain time point n, m,_…_ etc.

*average V*_*m,n*,…_ representing the average speed calculated for each time point

By normalizing the above mentioned parameters, we can confirm that reported significant differences are independent of the speed of each individual animal.

##### General parameters

Some rats demonstrated start hesitation or stopped and turned around after they entered the corridor. This behavior was frequently observed in the MFB lesion cohort starting from week 1 after 6-OHDA lesioning. For all the analyzed parameters, faulty or incomplete step cycles at the start or end of runs were deleted and the classification of runs was performed on at least three uninterrupted step cycles.

*Average speed*: Speed displayed during a recorded run from entering until leaving the walkway

*Proportion of slow moving rats*: The percentage of slow moving animals were plotted for each time point and treatment group. As discussed by Vlamings et al. (2007) and Koopmans et al. (2005) velocities between 20 and 50 cm/s are considered as slow walking. The speed at which an animal walks is very likely an indication of impaired motor abilities. Velocities below 20 cm/s are more attributable to explorative behavior, characterized by frequent pauses and accompanied by sniffing, thus all animals walking at <20 cm/s were excluded from the analysis of “average speed” and all other speed associated variables (Vlamings et al., 2007).

##### Dynamic paw parameters

*Step cycle*: Describes the time in seconds between two consecutive initial contacts of the same paw with the glass floor of the walkway and consists of the stand phase and swing phase:

1. *Stand phase*: Reflects the duration in seconds of contact of a paw with the glass floor.
2. *Swing phase*: Expresses the duration in seconds of no contact of a paw with the glass plate.

*Cadence*: Describes how many steps are taken within 1 min.

##### Coordination

*Stand index*: This is a measure for the speed at which a paw loses contact with the glass plate at the initiation of the swing phase.

*Print position*: This is the distance in cm between the position of the hind paw and the position of the previously placed front paw on the same side of the body (ipsilateral) and in the same step cycle. A positive value of the print position indicates that the hind paw is placed behind the front paw. A negative value of the print position indicates that the hind paw is placed in front of the front paw.

*Initial dual stance*: This is the first time in a step cycle of a front or hind paw that the contralateral front or hind paw also makes contact with the glass plate.

*Phase Dispersion*: This is a measure of inter-limb coordination and describes the temporal relationship between placement of two paws within a step cycle as described by the CatWalk™ XT software. Phase Dispersion is the moment of initial contact of a target paw expressed as a percentage of the step cycle time of an anchor paw, and is calculated as follows:

$$\begin{array}{l} \text{Phase Dispersion }\left[\%\right]=\frac{\text{I}{\text{C}}_{\text{target}}\left[s\right]-IC_{anchor}\left[s\right]}{\text{step cycl}{\text{e}}_{\text{anchor}}\left[s\right]}\times 100\% \end{array}$$

IC describes the initial contact in seconds for either the target or the anchor paw. The anchor paw is always the front paw for diagonal phase dispersion (RF-LH or LF-RH).

Data was analyzed with a paired samples *t*-test within treatment groups for each time point. Before statistical analysis was conducted, a Pearson's correlation analysis was conducted to investigate a possible association of the various phase dispersion variables with the average body speed.

##### Static parameters

*Stride length*: This is the distance between successive placements of the same paw.

*Print length*: This is the length in cm (horizontal direction) of the complete print. The complete print is the sum of all contacts with the glass plate.

*Print area*: This describes the surface area in cm^2^ of the complete print.

#### Drug induced rotations

Rotational testing was used to confirm DA cell loss in the SNc. Thirty days after 6-OHDA lesioning, rats were placed inside a clear cylinder (width 30 cm, height 33 cm) immediately after injection of 1 mg/kg Apomorphine, s.c. (Sigma-Aldrich, St Louis, MO, USA) dissolved in 50 μM ascorbic acid. Rats were recorded for 60 min for post-analysis. Full 360^−^degree turns away from the side of injection (contralateral) and toward the side of injection (ipsilateral) were counted manually while watching the recordings. Net contralateral rotations per minute were calculated by subtracting the number of full ipsilateral turns of 60 min from the number of full contralateral turns of 60 min and dividing the difference by 60 min. The cut-off for successful MFB lesions was set to ≥5 rotations/min.

#### Cylinder test-spontaneous forelimb use asymmetry test

The protocol used in this study was adapted from Jones and Schallert (1994). Animals were placed in a Plexiglas cylinder (30 cm high and 20 cm internal diameter) and were left alone to explore for 5 min while being recorded. Mirrors were placed so that animals could be filmed from all angles. An investigator blinded to the treatment group later recorded the number of times each forepaw was used to initiate weight-shifting movements including wall contact and landing. The use of both paws together was scored as both a left and a right paw movement. The results are presented as percentage of contralateral touches.

### Tissue processing and immunohistochemistry

Animals were culled on day 43 of the study with an anesthetic overdose of 150 mg/kg of sodium pentobarbitone i.p. and transcardially perfused with ice-cold 0.9% saline, followed by 400 ml of 4% paraformaldehyde in 0.1 M phosphate buffer pH 7.4. The brains were removed and post fixed overnight in 4% paraformaldehyde, then cryoprotected in 30% sucrose in 0.1 M phosphate buffer pH 7.2 for sectioning. Coronal sections were cut from the striatum through to the SNc on a sliding microtome set at 40 μm. Eight sets of sections were collected from each brain (320 μm between consecutive sections). Immunohistochemistry was performed on individual sets of rostro-caudal brain sections using an antibody against TH (1:1,000; Chemicon). Stereological quantification of TH positive neurons in the substantia nigra was performed using StereoInvestigator (MicroBrightfield, Williston, VT, USA) with optical fractionator probes set over an average of five coronal sections through the ipsilateral substantia nigra to the 6-OHDA injection site. Volume measurements of striatal regions with TH-positive fibers innervations of the ipsilateral striatum were performed on six coronal sections with StereoInvestigator (MicroBrightfield) Cavalieri Estimator probes using the lateral ventricle, corpus callosum and internal capsule to define the striatal borders.

### Statistical analysis

All gait parameters were analyzed using CatWalk™ XT software and expressed as mean ± s.e.m. The proportion of slow moving rats is expressed as a percentage of the total number of rats in a cohort. Data from the spontaneous forelimb use test (Cylinder test) is expressed as a percentage of contralateral touches ± s.e.m. and data from the rotational analysis is expressed as net contralateral turns per minute ± s.e.m. Two-way repeated measure ANOVA was used for the statistical evaluation of gait changes in the CatWalk™ XT test and for the spontaneous forelimb use test. Influence of time, treatment (sham MFB, sham striatal, MFB lesion, and striatal lesion) or time × treatment on the different CatWalk™ XT parameters or on the proportional use of the left forelimb was evaluated. The Greenhouse-Geisser correction was used if sphericity could not be assumed. For multiple comparisons test, depending on whether the Levene's test was significant (assumption of homogeneity violated) or not significant, the Dunnett's *post-hoc* or Tukey's *post-hoc* tests were used, respectively for cases where the interaction time × treatment effect was significant. When the interaction was not significant, influence of the treatment factor was independently analyzed. For the parameter Phase Dispersion a paired-samples *t*-test was used to analyze pairwise differences of diagonal or ipsilateral phase dispersion within each treatment group over time. Statistical significance for rotation testing was analyzed using one-way ANOVA and group differences were determined by the Tukey's *post-hoc* test or Dunnett's *post-hoc*.

The correlations of the number of neurons with positive immuno-staining for TH with gait parameters in the CatWalk™ XT test were evaluated by the Pearson's product correlation coefficient. Differences in the number of TH positive neurons in the substantia nigra for the different treatment groups were analyzed using one-way ANOVA and Tukey's *pots-hoc* test, respectively.

All statistical tests were performed with SPSS 23.0 statistical software (IBM, Armonk, NY, USA). A *p-*value of 0.05 or lower was considered statistically significant and all outliers beyond ± 2 *SD* were removed from the analysis. No difference was observed for any of the gait parameter or behavioral test measures when the MFB and striatal sham lesion animals were compared.

## Results

### Behavioral testing

#### Gait analysis

The gait of rats was evaluated with the CatWalk™ XT system prior to 6-OHDA lesioning (baseline testing) and then weekly after administration of either 6-OHDA or saline (Figure 1 and Table 1). Twenty-six variables showed significant differences at one or more-time points for the MFB lesion cohort compared to the sham cohort. Independency of results from interplay with velocity was achieved through normalization of previously reported speed-associated parameters step cycle, swing phase, stand phase, stride length, print position and cadence, against average speed. This ensured that any significant change observed was not due to the difference in walking speed, as reduced motivation in the MFB lesion cohort was observed frequently, but rather due to the impact of the manipulation on an animal's motor ability.

**Table 1:** Summary of gait parameters results.

**Parameter**	**Striatal lesion**	**MFB lesion**
Average speed	↔	↓ starting from week 1
Cadence	↔	↓ starting from week 1
Slow moving animals	↔	↑
Stride length	↔	↓ in all paws, starting from week 1
Initial dual stance	↑ in the LF on week 4	↑ in all paws, starting from week 1
Paw print position	↔	↑ in left and right paws
Step cycle:	↔	↓ in all paws
Stand phase		↔
Swing phase		↓ for LF, RF and LH
Stand index	↔	↑ in all paws. LF and RF starting from week 1; LH from week 2 to 5; RH from week 3 to 5
Phase dispersion	↓ of the LF-RH axis vs. RF-LH axis dispersion, starting from week 1	↓ of the LF-RH axis vs. RF-LH axis dispersion, starting from week 1
Print length	↔	↑ in RH, for weeks 2, 3, 4, and 6
Print area	↔	↑ in RH, from weeks 2 to 5

*Lesion groups are compared to their control (except for Phase dispersion parameter). Data shows: no significant differences observed (↔), significant increase observed (↑) and significant reduction observed (↓). LF, left front paw; RF, right front paw, LH, left hind paw and RH, right hind paw*.

##### Average speed

**Figure 2:**
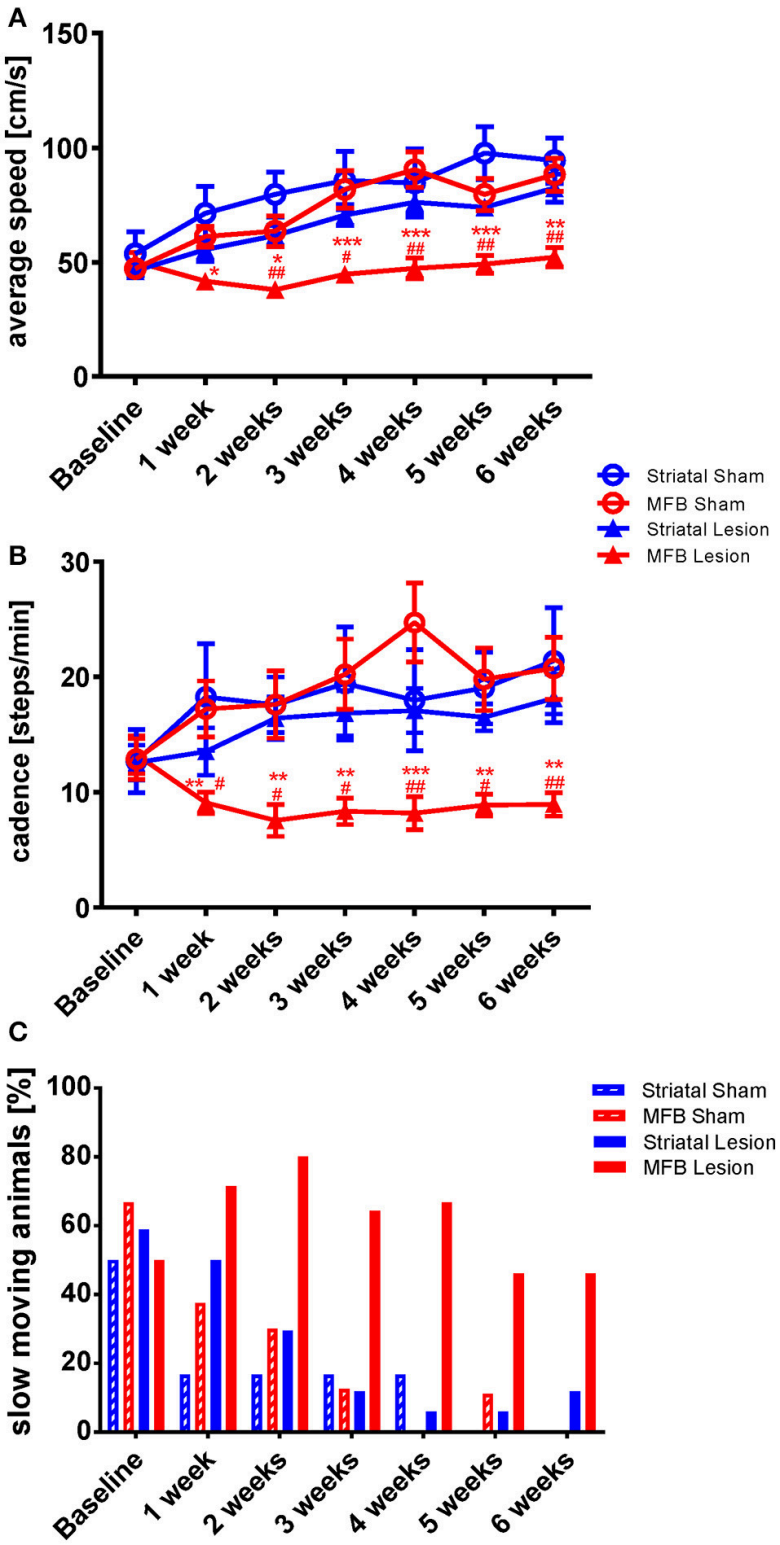
Kinetic variability of gait following MFB or striatal 6-OHDA lesions. Graphs demonstrating the inter-dependent variables **(A)** average speed of a run, **(B)** the total number of steps taken per minute (cadence) and **(C)** the percentage of slow moving rats within each cohort. Data shown as mean ± s.e.m **(A,D)** or percentage **(C)**. ^*^Indicates a significant difference on the lesion group when compared to its control (blue for striatal cohort and red for MBF cohort) and ^#^indicates differences of MFB lesion vs. striatal lesion animals. ^*^P ≤ 0.05; ^**^P < 0.01; ^***^P < 0.001 (Two-way repeated measures ANOVA with Tukey's or Dunnett's *post-hoc* multiple comparison).

While there was a steady increase in average speed from 1 week to another in the sham groups and in the striatal cohort, the average speed hardly changed in the MFB lesion cohort over the study period (Figure 2A). A two-way repeated measures ANOVA examining the interaction of time × treatment showed a significant effect [*F*_(18, 284)_ = 3.306, *p* < 0.001]. *Post-hoc* multiple comparisons shows a significant reduction on the average speed in the MFB lesion group when compared with the sham MFB group starting at week 1 post lesion until the end of the experiment, as well as a significant difference when compared to striatal lesion cohort starting from week 1 (Figure 2A). No significant differences where observed for the striatal lesion group when compared to control.

##### Cadence

Cadence is a parameter that describe the number of steps per minute. It is a gait parameter strictly related with average speed and it is expected that animals that show a higher average speed will, show a higher cadence. The MFB lesion cohort showed a significant reduction of cadence as shown by the interaction of time × treatment effect. [*F*_(18, 284)_ = 2.136, *p* = 0.02]. *Post-hoc* multiple comparisons indicated a significant decrease in cadence in the MFB lesion group when compared with the sham MFB group starting 1 week post-lesion until the end of the experiment as well as a significant difference when compared to the striatal lesion group starting from week 1 (Figure 2B). No significant differences where observed for the striatal lesion group when compared to its control group.

We also observed that the percentage of slow moving rats declined steadily in the sham and striatal lesion groups over the study period with both sham lesion groups reaching zero by 6 weeks post lesion (Figure 2C). The MFB lesion cohort, however, showed an increasing number of slow-moving individuals during the first 2 weeks. The percentage was then reduced, but still higher than the sham groups and the striatal lesion cohort during the whole experiment (Figure 2C).

##### Stride length

**Figure 3:**
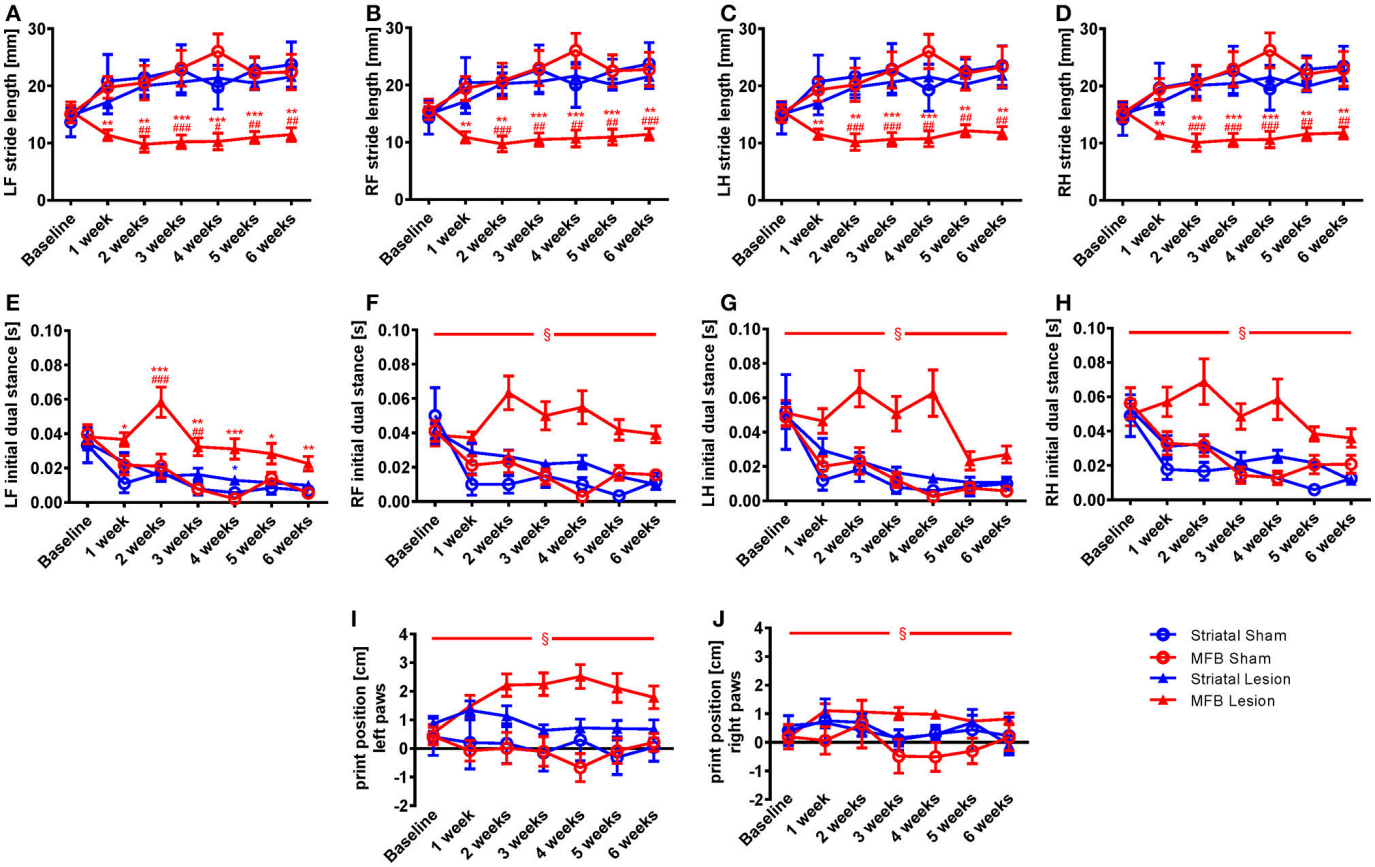
Stride length, initial dual stance and print position following MFB and striatal 6-OHDA lesioning. Graphs demonstration **(A–D)** the average stride lengths for each paw, **(E–H)** duration of simultaneous contact of hind or front paws with the walkway (dual stance), **(I–J)** the distance between the position of the hind paw and the position of the previously placed front paw on the same side of the body. LF, left front, RF, right front, LH, left hind, RH, right hind. Data shown as mean ± s.e.m. ^*^Indicates a significant difference on the lesion group when compared to its control (blue for striatal cohort and red for MBF cohort) and ^#^indicates differences of MFB lesion vs. striatal lesion animals. ^*^P ≤ 0.05; ^**^P < 0.01; ^***^P < 0.001 (Two-way repeated measures ANOVA with Tukey's or Dunnett's *post-hoc* multiple comparison when interaction time × treatment was significant). ^§^Indicates a significant influence of the treatment factor independently, when comparing the four different groups.

In order to further analyze the changes on the dynamic paw parameters observed in the MFB lesion animals, we studied the stride length parameter that describes the distance between successive placements of the same paw. Our data show that the normalized parameter stride length was significantly reduced for all paws in the MFB lesioned rats (Figures 3A–D). Significant effect of time × treatment interaction were observed at the left front [*F*_(18, 284)_ = 2.194, *p* = 0.05], the right front [*F*_(18, 284)_ = 2.126, *p* = 0.05], the left hind [*F*_(18, 284)_ = 2.114, *p* = 0.07], and the right hind [*F*_(18, 284)_ = 2.161, *p* = 0.005]. *Post-hoc* multiple comparisons showed a significant difference in the MFB lesion group when compared with the sham MFB group starting 1 week post-lesion until the end of the experiment as well as a significant difference when compared to the striatal lesion group starting from week 2 post-lesion for all the paws (Figures 3A–D). No significant differences where observed for the striatal lesion group when compared to the sham striatal control for any of the paws.

##### Initial dual stance

The initial dual stance refers to the period of time during a step cycle when both the contralateral front and hind paws are in contact with the walkway and support the animals body weight. It is a regularity measure and variability gives the appearance of limping and postural instability. A significant increase in the length of contact of all paws was noted in the MFB lesion cohort when compared to the other groups (Figures 3E–H). For the left front initial dual stance there was a significant effect of time × treatment [*F*_(18, 284)_ = 2.483, *p* = 0.001]. *Post-hoc* multiple comparisons showed a significant difference for the MFB lesion group when compared to the sham MFB group starting 1 week post-lesion until the end of the experiment (Figure 3E). Significant differences in dual stance were also detected for the left front paw at 2 and 3 weeks post-lesion when the MFB lesion cohort was compared to striatal lesion group. Curiously, a significant increase in the left front initial dual stance was observed for the striatal group when compared to its control on week 4 (Figure 3E).

For the other paws no significant interaction of time × treatment was detected, but a significant effect of treatment was independently observed for the right front [*F*_(3, 40)_ = 20.321, *p* < 0.001] (Figure 3F); the left hind [*F*_(3, 40)_ = 4.481, *p* = 0.011] (Figure 3G) and the right hind [*F*_(3, 40)_ = 4.843, *p* = 0.008] (Figure 3H).

##### Paw print position

The normalized parameter print position, a further regularity measure, showed an increase in paw position in the MFB lesion cohort when compared to other treatments (Figures 3I,J). While there was no significant effect of time × treatment there was a significant effect of treatment for both the left paws [*F*_(3, 40)_ = 4.644, *p* = 0.008] (Figure 3I) and the right paw [*F*_(3, 40)_ = 2.894, *p* = 0.049] (Figure 3J).

##### Step cycle

**Figure 4:**
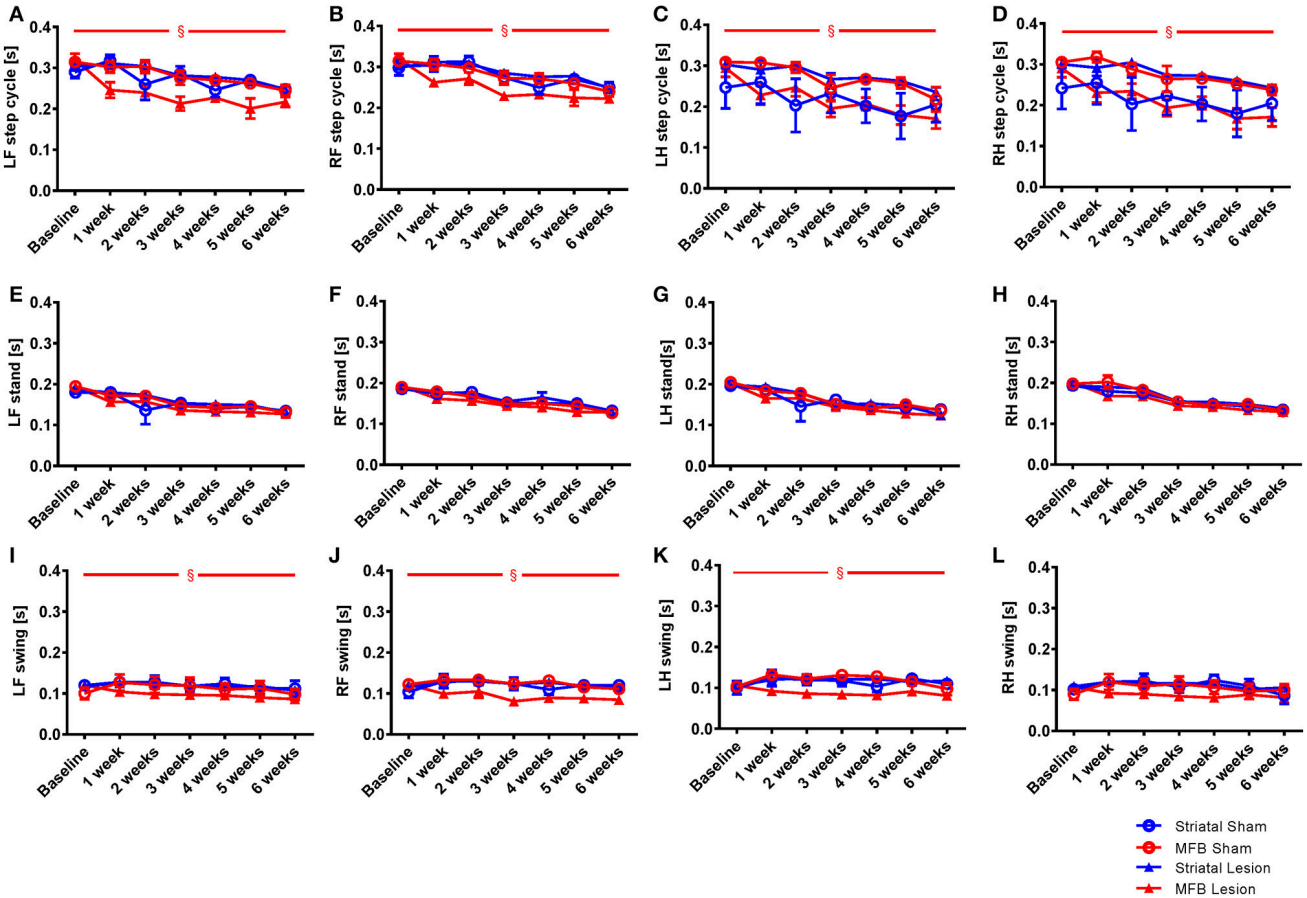
Length of step cycle and its components stand phase and swing phase following MFB or striatal 6-OHDA lesioning. Graphs demonstrating: **(A–D)** the time in seconds between two consecutive initial contacts of the same paw (step cycle) and consists of the stand and swing phase; **(E–H)** the duration in seconds of contact of a paw with the walkway (stand); **(I–L)** the duration in seconds of no contact for a paw with the walkway (swing). LF, Left front; RF, right front; LH, left hind; RH, right hind. Data shown as mean ± s.e.m. (Two-way repeated measures ANOVA). ^§^Influence of the treatment factor independently, when comparing the four different groups.

Step cycle describes the time between two consecutive initial contacts of the same paw with the glass floor of the walkway. When we examined the average duration of the step cycle for each treatment group, significant differences were only observed in the MFB lesion cohort, in which a decrease in average duration of step cycle was observed (Figures 4A–D). Our data showed no significant effect of time × treatment for any of the paw for the step cycle parameter but a significant effect was observed when treatment was studied independently for the left front [*F*_(3, 40)_ = 4.843, *p* = 0.006] (Figure 4A), the right front [*F*_(3, 40)_ = 2.801, *p* = 0.044] (Figure 4B), the left hind paw [*F*_(3, 40)_ = 3.801, *p* = 0.017] (Figure 4C) and the right hind paws [*F*_(3, 40)_ = 7.239, *p* = 0.01] (Figure 4D).

The step cycle is made up of two stages, the stand phase (Figures 4E–H), which describes the time of contact of a limb with the floor of the walkway, and the swing phase (Figures 4I–L), which describes the time of no contact of a limb with the floor. We found no effect of lesion over time for the stand parameter for any paw (Figures 4F–H). When we examined the swing phases (Figures 4I–L) we found no significant effect of time × treatment for any paw, but a significant effect of treatment for the left front [*F*_(3, 40)_ = 3.906, *p* = 0.017] (Figure 4K), the right front [*F*_(3, 40)_ = 2.632, *p* = 0.046] and the left hind paws. No effect of lesion was observed for the right hind paw (Figure 4L). For the striatal lesion cohort, no significant differences were observed for any aspect of the step cycle when compared to sham control.

##### Stand index

**Figure 5:**
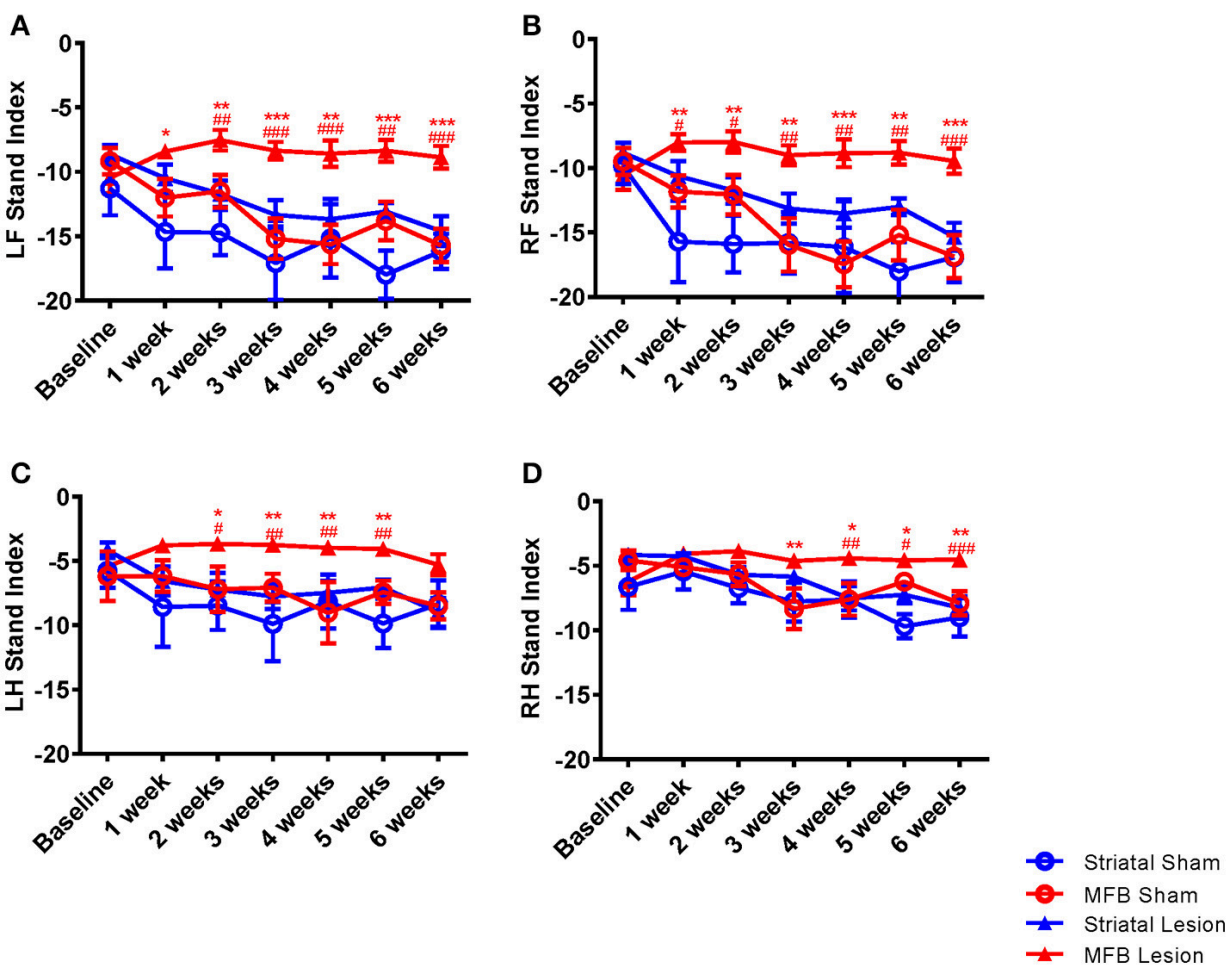
Graphs demonstrating the stand index following MFB or striatal 6-OHDA lesioning. **(A)** LF: left front, **(B)** RF: right front, **(C)** LH: left hind, and **(D)** RH: right hind. Data shown as mean ± s.e.m. ^*^Indicates a significant difference on the lesion group when compared to its control (blue for striatal cohort and red for MBF cohort) and ^#^indicates differences of MFB lesion vs. striatal lesion animals ^*^P ≤ 0.05; ^**^P < 0.01; ^***^P < 0.001 (Two-way repeated measures ANOVA with Tukey's or Dunnett's *post-hoc* multiple comparison).

The speed at which a paw loses contact with the walkway is represented in the parameter stand index (Figures 5A–D). It was noted that the lift-off of both front limbs of animals in the MFB lesion cohort was markedly faster than the sham cohort starting 1 week after 6-OHDA lesion and plateauing without noticeable change throughout the study period. Our data showed a significant interaction of time × treatment [*F*_(18, 284)_ = 2.647, *p* < 0.001] for the left front paw. *Post-hoc* multiple comparisons showed a significant difference in the stand index in the MFB lesion group compared to the MFB sham group from 1 week post-lesion as well as a significant difference compared to the striatal lesion group from week 2 post-lesion (Figure 5A). The right front stand index parameter also showed a significant interaction of time × treatment [*F*_(18, 284)_ = 2.780), *p* < 0.001]. *Post-hoc* multiple comparisons showed a significant difference in the stand index in the MFB lesion group compared with the sham MFB group from week 1 post-lesion as well as a significant difference compared to striatal lesion group starting from week 1 post-lesion (Figure 5B).

A similar observation was made for the hind paws (Figures 5C,D), however to a lesser extent than for the front paws. Left hind stand index parameter showed a significant interaction of time × treatment [*F*_(18, 284)_ = 5.108, *p* = 0.035]. *Post-hoc* multiple comparisons showed a significant difference in the MFB lesion group compared with the sham MFB group from 2 to 5 weeks post lesion as well as a significant difference compared to striatal lesion group starting from 3 to 5 weeks post lesion (Figure 5C).

The right hind stand index parameter also showed a significant interaction of time × treatment [*F*_(18, 284)_ = 3.257, *p* = 0.001]. *Post-hoc* multiple comparisons indicated a significant difference in the MFB lesion group compared with the sham MFB group from 3 to 5 weeks post lesion as well as a significant difference compared to striatal lesion group from week 4 post lesion (Figure 5D). For all paws, the striatal lesion cohort did not show any significant difference when compared to the striatal sham group.

##### Phase dispersion

**Figure 6:**
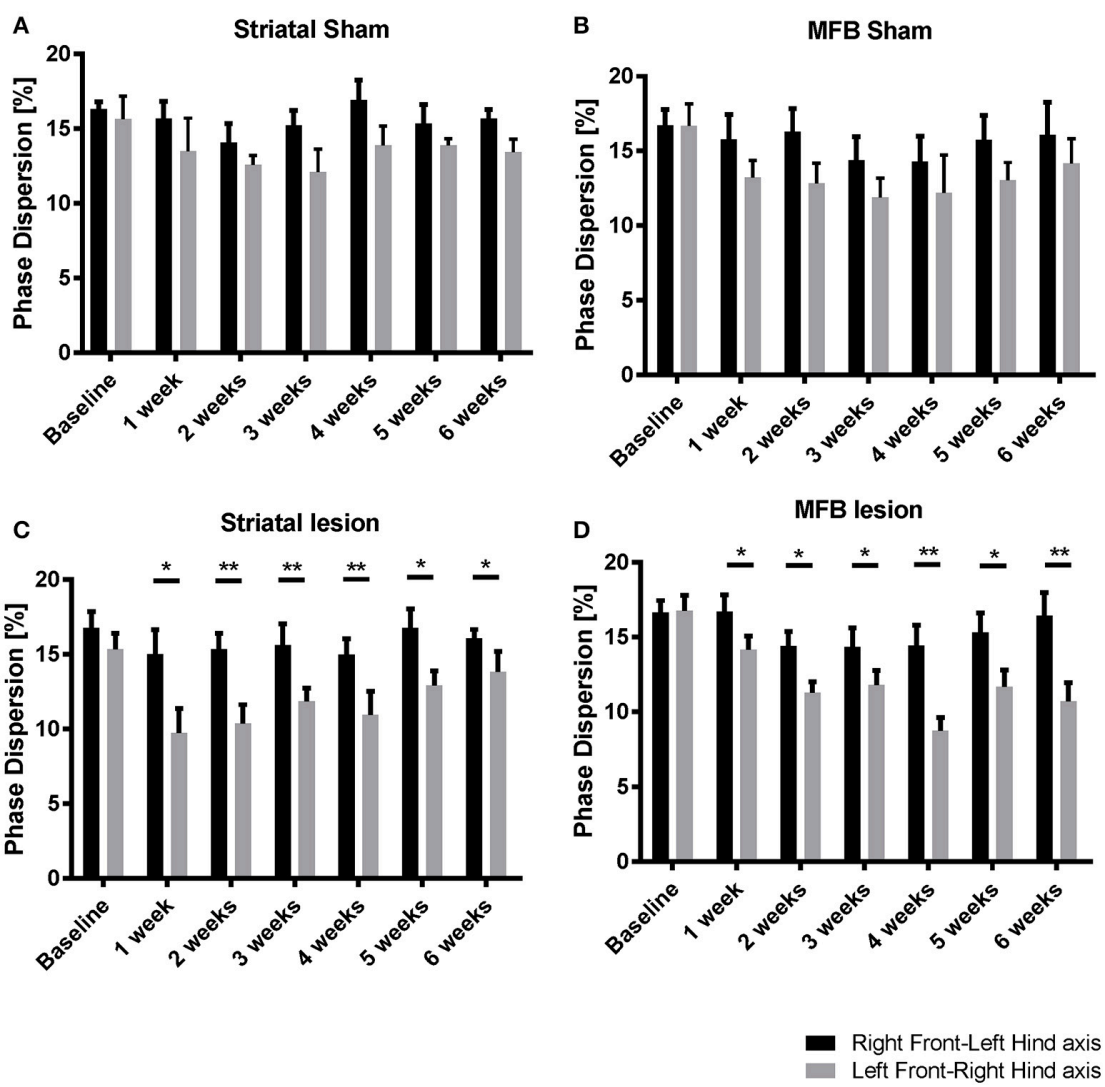
Diagonal phase dispersion alterations following MFB or striatal 6-OHDA lesions. **(A)** Striatal sham cohort, **(B)** MFB sham cohort, **(C)** Striatal lesion group, and **(D)** MFB lesion group. Data shown as mean ± s.e.m. ^*^P ≤ 0.05; ^**^P < 0.01 (paired-sample Student *t*-test).

To identify the effect of 6-OHDA lesioning on inter-limb coordination, diagonal phase dispersion as well as ipsilateral phase dispersion were compared within treatment groups over time (Figures 6A–D). In a perfectly balanced walking pattern it is not expected to find any differences when one side of the body or body axis is compared with the opposite side or axis. Any deviation from a coordinated walking pattern should be reflected by changes in one or both of these measures. There was no significant difference when comparing the mean ipsilateral phase dispersion values for Right Front- Left Hind axis with Left Front-Right Hind axis within treatment groups over time (data not shown). However, there were significant differences in the diagonal phase dispersion values at all the time points for both the striatal (Figure 6C) and the MFB (Figure 6D) lesion groups. In both cohorts, Left Front-Right Hind axis showed a significant reduction when compared to the Right Front- Left Hind axis starting at week 1 post lesion indicating a deviation on the coordinated walking pattern. No significant differences were observed in the sham groups.

##### Print length and area

**Figure 7:**
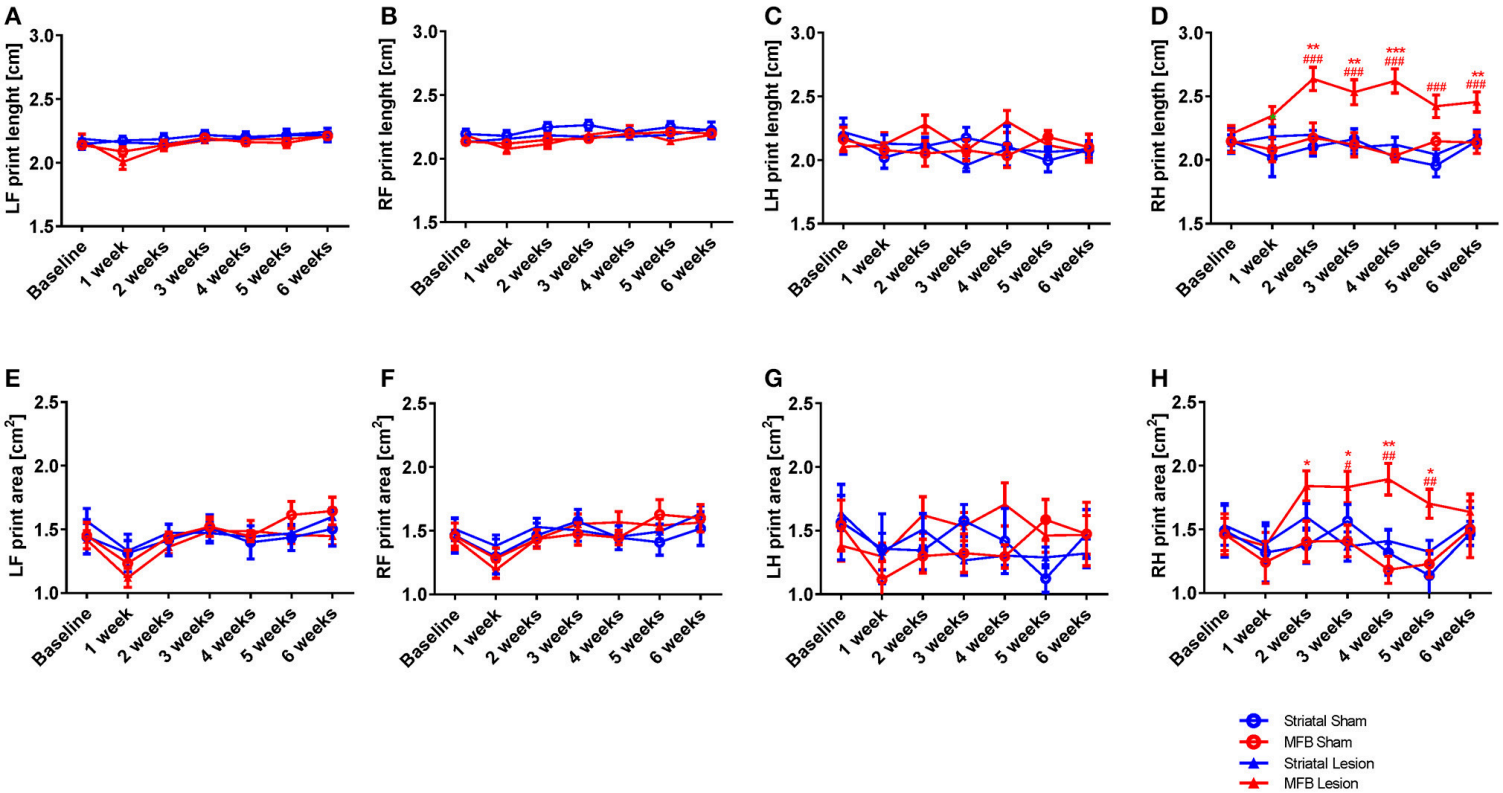
Print length and print area following MFB and striatal 6-OHDA lesioning. Graph demonstrate **(A–D)** the length of the complete prints and **(E–H)** the print area. Data shown as mean ± s.e.m. ^*^Indicates a significant difference on the lesion group when compared to its control (blue for striatal cohort and red for MBF cohort) and ^#^indicates differences of MFB lesion vs. striatal lesion animals. ^*^P ≤ 0.05; ^**^P < 0.01; ^***^P < 0.001 (Two-way repeated measures ANOVA with Tukey's or Dunnett's *post-hoc* multiple comparison).

The print length and print area parameters are calculated on the base of the sum of all contacts of a paw with the glass floor. The more weight an animal puts onto a limb, the greater the contact area or print length value. Our data showed a significant increase in the right hind print length of animals in the MFB cohort. Two-way repeated measures ANOVA shows a significant interaction of time × treatment [*F*_(18, 284)_ = 1,824, *p* = 0.024]. *Post-hoc* multiple comparisons showed a significant difference in the print length of the MFB lesion group when compared with the MFB sham group for weeks 2, 3, 4, and 6 post lesion as well as a significant difference when compared to the striatal lesion group starting from 2 weeks post lesion (Figure 7D). However, no effect of lesion over time was observed for the other paws (Figures 7A–C).

Similarly, the print area parameter showed no significant interaction of time × treatment for any of the paws (Figures 7E–G) except for the right hind [*F*_(18, 284)_ = 1.558, *p* = 0.042] in where an increase in print area was observed in the right hind limb of MFB lesioned animal. *Post-hoc* multiple comparisons showed a significant difference in the MFB lesion group when compared with the MFB sham group from 2 to 5 weeks post lesion as well as a significant difference when compared to the striatal lesion group from 3 to 5 weeks post lesion (Figure 7H).

For all paws, the striatal lesion cohort did not show any difference in print area or length compared to the striatal sham group.

#### Spontaneous exploratory forelimb use test (cylinder test)

**Figure 8:**
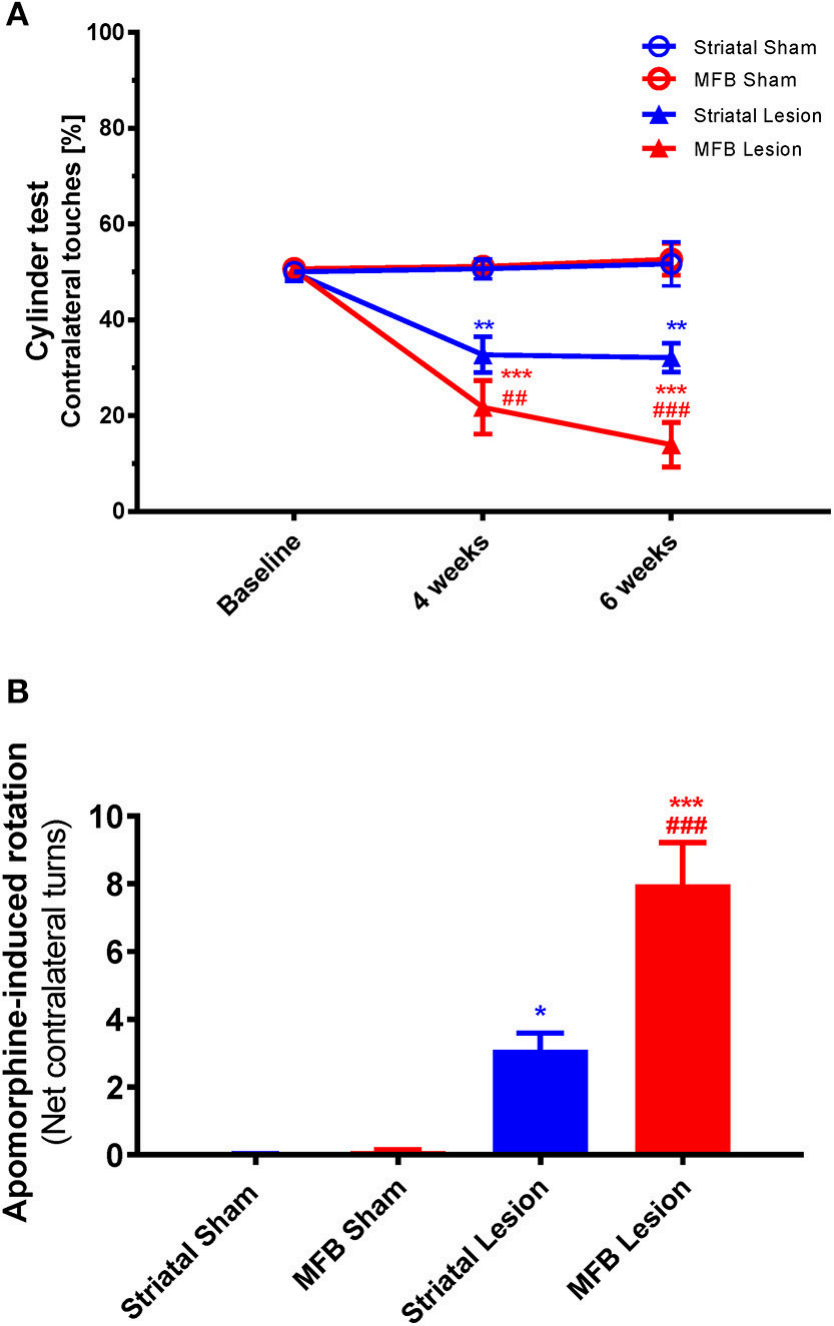
Cylinder test and Apomorphine-induced rotation following MFB and striatal 6-OHDA lesioning. **(A)** Percentage contralateral touches. **(B)** Net contralateral turns per minute. Data shown as mean ± s.e.m. ^*^Indicates a significant difference on the lesion group when compared to its control (blue for striatal cohort and red for MBF cohort) and ^#^indicates differences of MFB lesion vs. striatal lesion animals. ^*^P ≤ 0.05; ^**^P < 0.01; ^***^P < 0.001 [Two-way repeated measures ANOVA with Tukey's *post-hoc* multiple comparison for **(A)**, and one-way ANOVA with Tukey's *post-hoc* for **(B)**].

The cylinder test is a measure of sensorimotor forelimb function and was used as a reference to quantify the asymmetry of forelimb use in 6-OHDA lesioned animals compared to the sham-operated animals. We observed a significant reduction in use of the contralateral front paw in both the striatal and the MFB lesion cohorts 4 and 6 weeks after the 6-OHDA lesioning (Figure 8A). Two-way repeated measures ANOVA showed a significant interaction of time × treatment [*F*_(4, 40)_ = 24.336, *P* < 0.001] and *post-hoc* multiple comparisons showed a significant difference in the MFB lesion group compared with the MFB sham and the striatal lesion groups at 4 and 6 weeks post lesion. The striatal lesion cohort also demonstrated a significant reduction in contralateral forelimb use when compared to the striatal sham group.

#### Drug-induced rotations

Drug-induced rotational behavior is a robust indicator of unilateral dopaminergic 6-OHDA lesioning. The rotational response to Apomorphine was examined 4 weeks after 6-OHDA lesioning (Figure 8B). The number of Apomorphine-induced contralateral rotations during a 60 min period for animals in the sham MFB, sham striatal, striatal lesion and MFB lesion cohorts was analyzed manually. Four weeks after 6-OHDA, highly significant rotational behavior was observed in the MFB lesion cohort when compared to sham MFB group (7.99 rotations/min and 0.10 rotations/min, respectively; *P* < 0.001). The striatal cohort also showed a significant increase in the rotation response after been challenged with Apomorphine when compared to its control (3.10 rotations/min, and 0.03 rotations/min; *P* < 0.04).

### Quantification of dopaminergic neuronal degeneration following 6-OHDA lesioning

**Figure 9:**
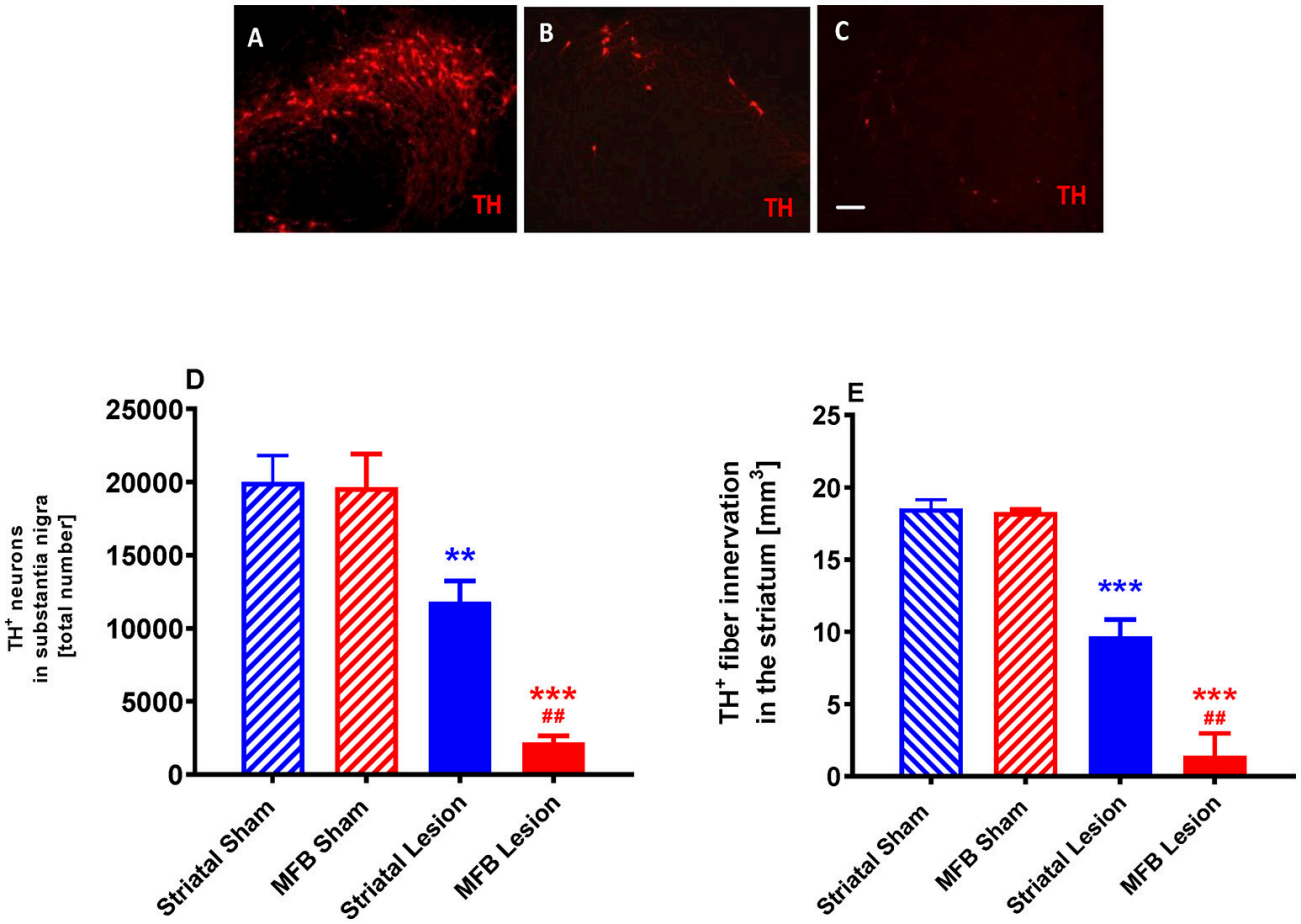
Quantification of dopaminergic neuronal degeneration following 6-OHDA lesioning. Representative pictures of TH-positive neurons in the SNc in **(A)** sham animals, **(B)** striatal lesion group **(B,C)** MFB lesion group. Quantification of **(D)** TH-positive neurons in the SNc and **(E)** level of TH immunoreactivity in the striatum **(E)**. Data shown as mean ± s.e.m. ^*^Indicates a significant difference on the lesion group when compared to its control (blue for striatal cohort and red for MBF cohort) and ^#^indicates differences of MFB lesion vs. striatal lesion animals. ^**^P < 0.01; ^***^P < 0.001 (one-way ANOVA with Tukey's or Dunnett's *post-hoc*).

Stereological quantification revealed a significant reduction in the total number of tyrosine hydroxylase (TH) -positive neurons in the SNc of either the MFB or the striatal lesion cohorts (MFB: 2,199 ± 457 TH-positive cells, *P* < 0.001; Striatal: 11,803 ± 1,438 TH-positive cells, *P* < 0.001) when compared to sham groups (19,654 ± 2,246 and 20,015 ± 1,785, respectively) 6 weeks post lesion (Figures 9A–D). No significant differences in the number of TH-positive neurons was observed between the two sham groups.

We also observed a significant reduction in the level of TH immunoreactivity in the striatum of animals in both the MFB lesion (1.41 ± 1.55 mm^3^, *P* < 0.001) and the striatal lesion cohorts (9.66 ± 1.19 mm^3^, *P* < 0.001) when compared to MFB sham (18.27 ± 0.2 mm^3^) and striatal sham (18.52 ± 0.63 mm^3^) groups 6 weeks after 6-OHDA lesioning (Figure 9E). No difference in the level of TH immunoreactivity was observed in the striatum between the two sham groups.

### Correlation of gait parameters with TH-positive neuronal cell loss

As we described on the results, the MFB lesion cohort present alterations in different gait parameters analyzed. In order to further analyzed this point, a correlation study was conducted to confirm the relationship between TH neuronal cell loss of the SNc and gait dysfunction in the MFB cohort and its control. A Pearson's correlation coefficient (*R*) was calculated for the average value of 27 significant gait parameters and the residual number of TH-positive neurons in the SNc of the MFB lesion cohort 6 weeks after lesioning. A strong correlation was detected for average speed, cadence, swing (left hind), and stride length (right front). In contrast, a moderate correlation was identified for 18 gait analysis parameters with no correlation for the remaining 6 parameters (Table 2 and Supplementary Figures 1–3).

**Table 2:** Correlation of motor deficits with the number of tyrosine hydroxylase positive neurons in the substantia nigra in the MFB lesion cohort and MFB sham cohort 6 weeks after lesioning.

**Parameter**	**Pearson correlation coefficient**	***P*-value**	**Parameter**	**Pearson correlation coefficient**	***P*-value**
			**Step Cycle [s]**		
Run Duration [s]	−0.529	0.008^**^	RF	−0.552	0.006^**^
			RH	−0.525	0.012^*^
			LF	−0.592	0.004^**^
Average Speed [cm/s]	0.635	0.001^***^	LH	−0.590	0.008^**^
			**Stand [s]**		
Cadence [steps/min]	0.608	0.002^**^	RF	−0.489	0.021^*^
			RH	−0.528	0.014^*^
			LF	−0.555	0.007^**^
			LH	−0.420	0.065
**Initial Dual Stance [s]**			**Swing [s]**		
RF	−0.456	0.013^*^	RF	−0.442	0.035^*^
RH	−0.291	0.133	RH	−0.556	0.006^**^
LF	−0.290	0.127	LF	−0.593	0.004^**^
LH	−0.305	0.115	LH	−0.665	0.001^***^
**Print Length [cm]**			**Stride Length [cm]**	
RF	0.176	0.421	RF	0.602	0.002^**^
RH	−0.407	0.054	RH	0.577	0.004^**^
LF	0.149	0.498	LF	0.557	0.006^**^
LH	0.152	0.510	LH	0.505	0.016^*^

*Strong correlation R ≥ 0.6; moderate correlation 0.6 > R > 0.3; weak correlation R > 0.3*.

* P ≤ 0.05;

** P < 0.01;

*** *P < 0.001 (Pearson's correlation coefficient)*.

## Discussion

Clinically, about 20% of PD patients over the age of 80 have parkinsonism-associated gait disturbances such as bradykinesia, hyperkinesia, increased double support duration, flat foot strike, reduced foot lifting as well as start hesitation and freezing of gait (Ebersbach et al., 1999; Hausdorff et al., 2003; Bloem et al., 2004; Jankovic, 2008; Hausdorff, 2009; Kwon et al., 2017). It is important for successful animal models of PD to exhibit motor symptoms, such as gait dysfunction that correspond to those experienced by human patients. In this study, we identified 21 different gait parameters that were significantly altered in the MFB 6-0HDA lesion rat model. Some of these parameters are considered interdependent: average speed, cadence and proportion of slow moving rats are all relative to the factor “time,” making them interdependent. Similarly, stand phase and swing phase are the two components of which the variable step cycle is made up from. However, even if taking these interdependencies into account, there were still more than 20 truly independent variables that show significant changes after 6-OHDA lesion, some as early as 1 week after the lesion. This suggests that gait analysis can provide a clinically relevant, low impact method of testing functional impairment.

The greatest impact of 6-OHDA lesioning could be seen with the average walking speed and cadence. Our data indicated that the overall walking speed and cadence of the MFB lesioned animals steadily declined when compared to the other groups. This data is reinforced with the percentage of slow moving rats' greatest in the MFB lesion cohort. Slow movement was defined as an average speed of 20–50 cm/s (Koopmans et al., 2005; Vlamings et al., 2007). Throughout the study period, 50–80% of the MFB group fell within this category, while sham groups and striatal lesion cohort showed a gradual increase of slow moving animals. Diminution of walking speed in different PD rat models have been previously reported (Vlamings et al., 2007; Hsieh et al., 2011). Interestingly, the alteration on average speed, cadence and the percentage of slow moving animals in the MFB lesion group was observed from the first week after the 6-OHDA lesion until the end of the experiment.

Our results also showed an alteration in the stride length parameter in the MFB lesion cohort. These animals demonstrated a significant reduction in stride length when compared to the MFB sham group and the striatal lesion cohort for all four limbs. This parameter indicates successive placement of the same paw and the reduction observed in the MFB lesion cohort is an indication of gait abnormalities similar to those observed in humans. Both initial dual stance and print positions, other measurement related with the initial step cycle, were significantly altered in the MFB lesion cohort further indicating gait abnormalities in this group. Interestingly, the alteration observed in the initial dual stance peaked at 2 weeks post lesion. The biological impact of this finding is difficult to determine without specifically investigating the temporal profile of TH+ neuronal cell loss. However, we suggest that this alteration reflects the full development of the nigrostriatal lesion.

Interestingly, we observed a significant increase in the stand index parameter for all four limbs of the MFB lesion cohort when compared to the MFB sham groups and the striatal cohort. This parameter is a measure for the speed at which a paw loses contact with the glass plate during one-step cycle. As previously mentioned, the MFB lesion cohort showed a significant reduction of average speed but a higher value of paw speed thus indicating an alteration in the footfall pattern and abnormalities in gait. Our data also showed that MFB lesion animals had a significantly higher value of right hind print length and print area starting from 2 weeks post lesion when compared to the MFB sham group and the striatal cohort. This value indicates a compensatory effect on the gait patter of those animals.

The effect of an MFB 6-OHDA lesion on inter-limb coordination was investigated by the parameter diagonal phase dispersion as well as ipsilateral phase within treatment groups over time. While no differences were found for the ipsilateral phase dispersion in either of the treatment groups, a clear impact on diagonal phase dispersion was observed. The most common footfall pattern for animals in this study was to alternate “Ab” with the following sequence: left front—right hind—right front—left hind. Thus, any coordination deficit would naturally show up in the diagonal phase dispersion measure, which reflects exactly this footfall pattern, rather than in a parameter that represents an alternating pattern “Aa” with a paw sequence of right front—right hind—left front—left hind, such as ipsilateral phase dispersion. Interestingly, the impairment of coordination of the diagonal axis seemed to be more prominent in the striatal lesion cohort compared to the MFB lesion cohort. This observation is the more surprising when looking at the results from the step-cycle analysis. The Swing phase for the right front/left hind axis of the MFB lesion cohort was significantly altered compared to its control, which supports the findings of the parameter diagonal phase dispersion. Yet, there were no measurable changes to any aspect of the step cycle for the striatal lesion cohort despite the significant changes to the diagonal phase dispersion in this group. Similarly, other parameters of coordination such as initial dual stance, stand index and print position show no or only minor changes in the striatal lesion group when compared to sham.

In the MFB lesion cohort all except for one observed gait parameter disturbance paralleled symptoms of advanced human PD. Slower average speed in combination with reduced cadence and significantly altered swing phase for the right front/left hind body axis may reflect bradykinesia (Jankovic, 2008; Turnbull and Fitzsimmons, 2009; Kwon et al., 2017). The altered stand index, stride length and print position reflects the typical small, shuffling steps observed in patients (Ebersbach et al., 1999; Hausdorff et al., 2003). This combination of measurements also suggests a reduced toe to ground clearance, as it is common in slower movements with shorter steps. These results are in accordance with those previously reported by Hsieh et al. (2011). Increased initial dual stance times reflect postural instability and prolonged double support duration in humans. While a significantly larger print area and print length was observed in the rat model, representing flat foot strikes in human patients, both of these parameters was increased only in the right-hind limb in our model, which was actually the non-impaired body side. Thus, this finding might be a compensatory measure rather than a true symptom. While it is commonly known that PD patients walk with an increased number of steps per minute (Hausdorff et al., 2003; Bloem et al., 2004; Hausdorff, 2009), some studies suggested that cadence is actually reduced in an early stages of PD (Pistacchi et al., 2017). Our results may therefore correlate with the response of cadence see in early stages of PD with a reduction in cadence observed in the MFB lesion cohort from 1 week post lesion. Finally, no recovery of function was observed in this study. DA terminal compensatory re-sprouting and motor function recovery has been reported to occur from 10 weeks onward following a MFB 6-OHDA lesion (Stanic et al., 2003, 2004). Our observation that gait impairment did not improve during the initial 6 week post lesion is in accordance with this timeframe and with previous publications (Hsieh et al., 2011; Zhou et al., 2015; Baldwin et al., 2017).

In contrast to the MFB lesion cohort, we observed limited alteration of gait function in the striatal lesion cohort. Although we observed a minimal gait disturbance in this group with increased initial dual stance on the left front paw at 4 weeks post lesion and phase dispersion alterations from week 1 to week 6, these observations did not correlate with the functional alterations observed in the cylinder test and with rotational analysis. The limited gait dysfunction observed in the striatal lesion cohort may be due to the lower level of DA cell loss (39.83%) observed in the striatal lesion cohort compared to the MFB lesion cohort (88.79%) at 6 weeks post lesion.

Further supporting this, we observed a significant correlation between TH neuronal cell loss in the SNc and gait dysfunction measured over 27 parameters. The MFB lesioned cohort showed an average cell loss of >90% when compared to MFB sham with these animals showing a higher disturbance on gait parameters. Overall, we observed 4 of 27 gait analysis parameters with a strong correlation, and 18 parameters showing a moderate correlation with the number of TH positive neurons in animals with MFB lesioned animals at 6 weeks post lesion. As expected, our correlation data indicates a clear interdependency between cell loss and gait disturbances, with higher cell loss resulting in greater disturbance. This indicates that gait analysis is a reliable tool to identify the severity of the DA depletion in a 6-OHDA rat model. While a correlation between TH cell loss and gait disturbance has already been described in mice (Wang et al., 2012) and rats (Vlamings et al., 2007; Hsieh et al., 2011; Baldwin et al., 2017) to the best of our knowledge, our data provides a more extensive and definitive correlation between DA depletion and alterations in the different stages of gait. However, a point to take into consideration is the possible effect of strain differences. It is known that behavioral differences are observed between the most common rat strains, Wistar, Sprague-Dawley, and Long-Evans (Cavigelli et al., 2013). Thus, the data we are reporting is this study can only be applied for the Wistar strain.

In conclusion, we have demonstrated that gait analysis is a reliable method for early detection of motor deficiencies in a MFB lesion model of PD potentially allowing the detection of motor function impairment earlier than with rotational analysis. The gait deficiencies observed in the MFB 6-OHDA lesion resemble those experience by PD patients and the use of gait may provide a valuable tool for the evaluation of future therapeutic strategies for PD.

## Author contributions

DvH performed the experiments; DvH and JB analyzed the data; DvH, JB, and BC prepared the manuscript.

### Conflict of interest statement

The authors declare that the research was conducted in the absence of any commercial or financial relationships that could be construed as a potential conflict of interest.
