# Supplementary material for: Gait Analysis for Early Detection of Motor Symptoms in the 6-OHDA Rat Model of Parkinson's Disease
Source: Front Behav Neurosci. 2018 Mar 6;12:39. doi: 10.3389/fnbeh.2018.00039 (PMC5845681; doi:10.3389/fnbeh.2018.00039)
Supplement: Supplementary file 1 [file DataSheet1.docx]

Supplementary Material

**Gait Analysis for Early Detection of Motor Symptoms In The 6-OHDA Rat Model of Parkinson’s Disease**

**Jordi Boix^1^_,_ Daniela von Hieber^1^ and Bronwen Connor^1^***

*** Correspondence:** Associate Professor Bronwen Connor, [b.connor@auckland.ac.nz](mailto:b.connor@auckland.ac.nz)

# Supplementary Figures


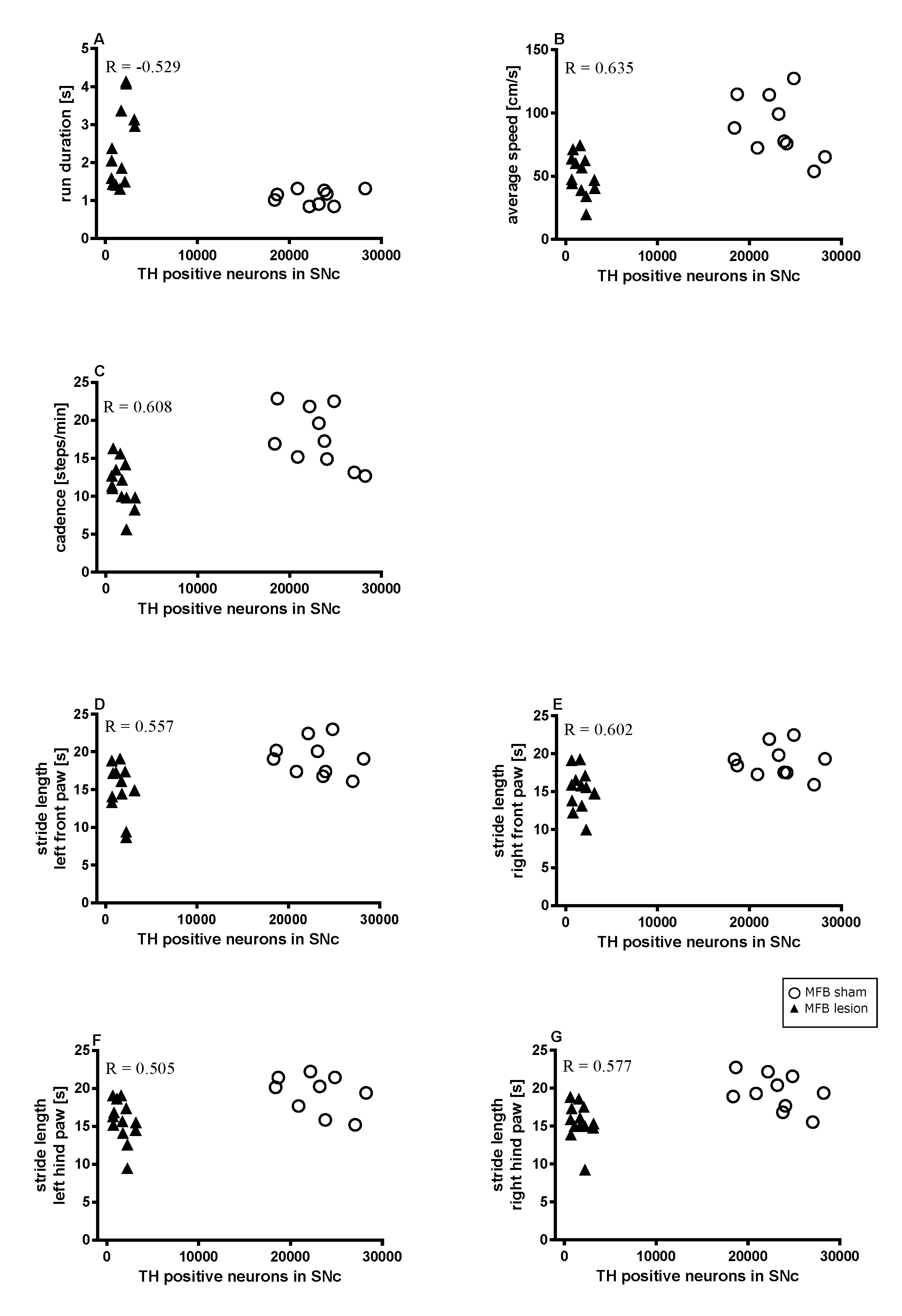


**Supplementary Figure 1. Correlation of kinetic parameters and dopaminergic depletion**. Graphs showing: (A) LF print length, (B) RF print length, (C) LH print length, (D) RH print length, (E) LF step cycle, (F) RF step cycle, (G) LH step cycle and (H) RH step cycle. Data shown: ▲ MFB cohort and MFB sham cohort (Pearson’s product correlation coefficient).


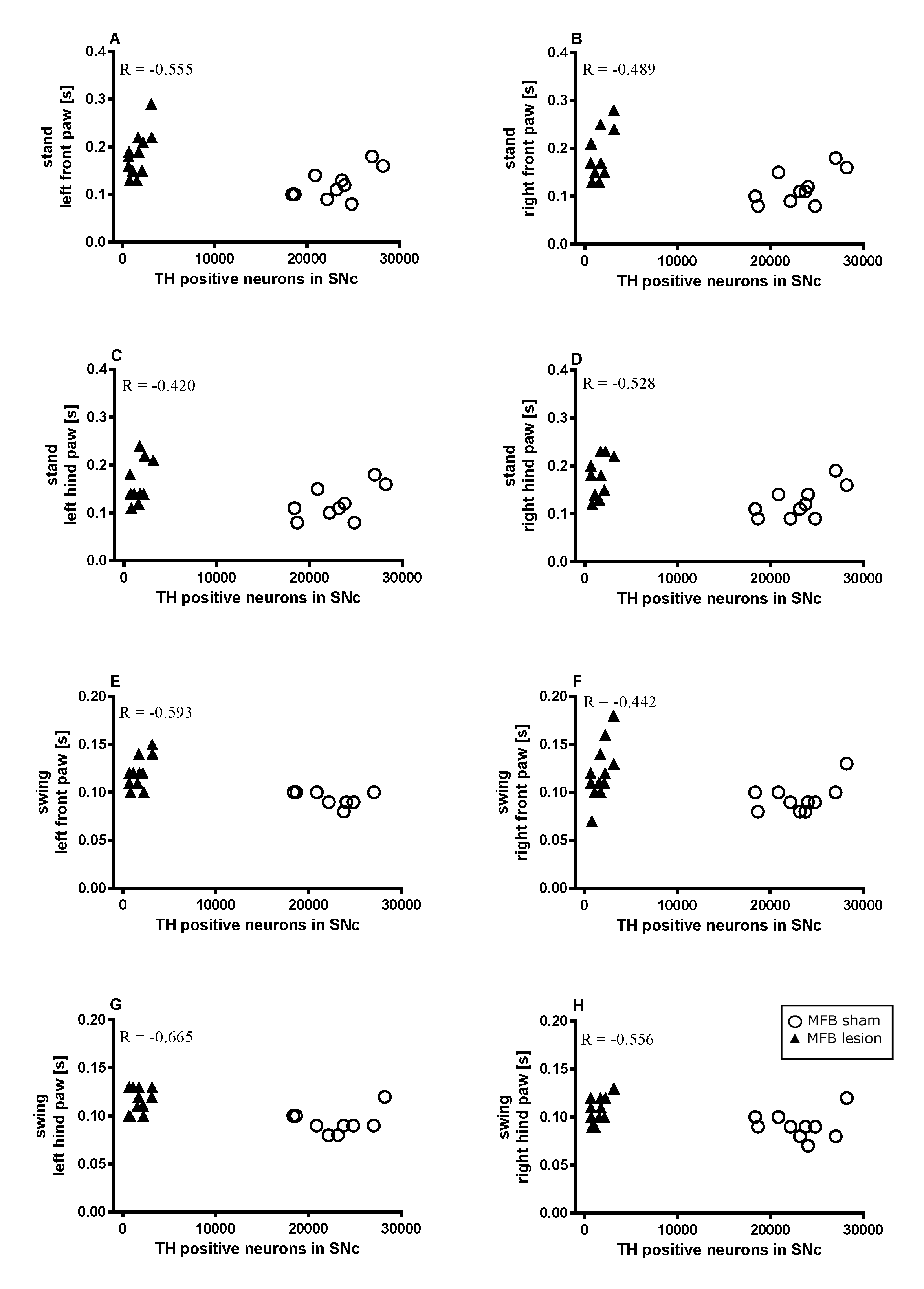


**Supplementary Figure 2. Correlation of print length and step cycle parameters and dopaminergic depletion**. Graphs showing: (A) LF print length, (B) RF print length, (C) LH print length, (D) RH print length, (E)LF step cycle, (F) RF step cycle, (G) LH step cycle and (H) RH step cycle. Data shown: ▲ MFB cohort and MFB sham cohort (Pearson’s product correlation coefficient).

**
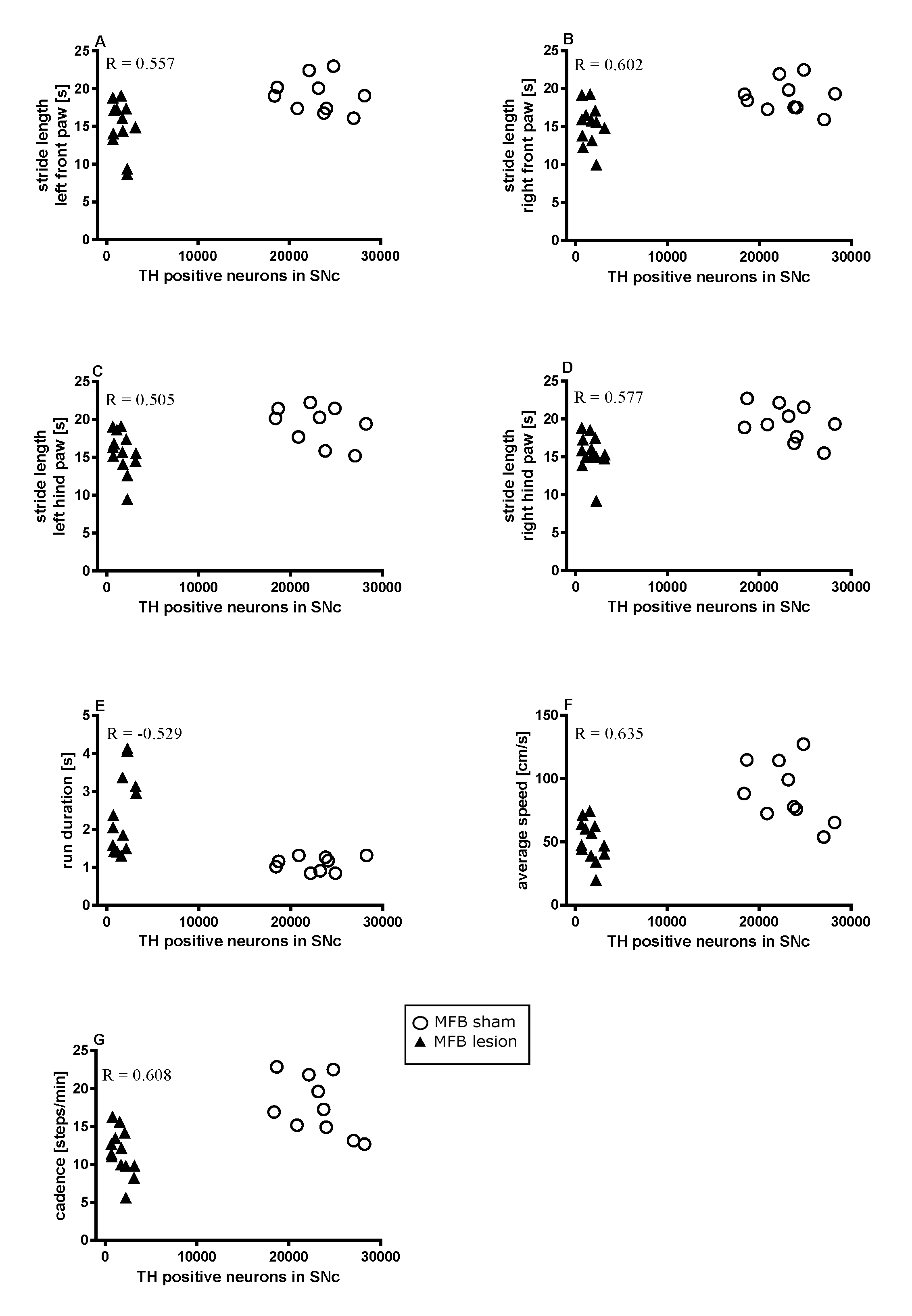
**

**Supplementary Figure 3. Correlation of step cycle components (stand and swing) and dopaminergic depletion**. Graphs showing: (A) LF stand phase, (B) RF stand phase, (C) LH stand phase, (D) RH stand phase, (E)LF swing phase, (F) RF swing phase, (G) LH swing phase and (H) RH swing phase. Data shown: ▲ MFB cohort and MFB sham cohort (Pearson’s product correlation coefficient).
